# 2,7-Dibromo-9,9-bis­[(pyridin-1-ium-4-yl)meth­yl]fluorene dinitrate

**DOI:** 10.1107/S1600536809054828

**Published:** 2010-01-09

**Authors:** Fang-Fang Jian, Shan-Shan Zhao, Huan-Mei Guo, Yu-Feng Li, Pu-Su Zhao

**Affiliations:** a Microscale Science Institute, Weifang University, Weifang 261061, People’s Republic of China; b New Materials and Function Coordination Chemistry Laboratory, Qingdao University of Science and Technology, Qingdao 266042, People’s Republic of China

## Abstract

In the title compound, C_25_H_20_Br_2_N_2_^2+^·2NO_3_^−^, the cation lies on a twofold rotation axis which imposes disorder of the dibromo­fluorene unit. In addition, the unique nitrate anion is disordered over two general sites of equal occupancy. The crystal structure is stabilized by inter­molecular N—H⋯O hydrogen bonds.

## Related literature

For applications of bipyridine derivatives, see: Varughese & Pedireddi (2005 ▶, 2006 ▶); Pedireddi & Lekshmi (2004 ▶); Friscic & MacGillivray (2005 ▶).
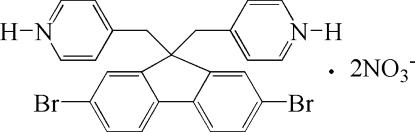


## Experimental

### 

#### Crystal data

C_25_H_20_Br_2_N_2_^2+^·2NO_3_^−^
*M* *_r_* = 632.27
Orthorhombic, 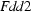

*a* = 14.874 (3) Å
*b* = 33.592 (7) Å
*c* = 10.720 (2) Å
*V* = 5356.2 (18) Å^3^
*Z* = 8
Mo *K*α radiation
μ = 3.07 mm^−1^
*T* = 293 K
0.25 × 0.20 × 0.18 mm

#### Data collection

Bruker SMART CCD diffractometer
Absorption correction: multi-scan (*SADABS*; Sheldrick, 1996 ▶) *T*_min_ = 0.514, *T*_max_ = 0.608
12890 measured reflections
3053 independent reflections
1355 reflections with *I* > 2σ(*I*)
*R*_int_ = 0.065

#### Refinement

*R*[*F* ^2^ > 2σ(*F* ^2^)] = 0.041
*wR*(*F* ^2^) = 0.114
*S* = 0.91
3053 reflections
238 parameters
77 restraints
H atoms treated by a mixture of independent and constrained refinement
Δρ_max_ = 0.20 e Å^−3^
Δρ_min_ = −0.18 e Å^−3^
Absolute structure: Flack (1983 ▶), 1443 Friedel pairs
Flack parameter: 0.002 (14)

### 

Data collection: *SMART* (Bruker, 1997 ▶); cell refinement: *SAINT* (Bruker, 1997 ▶); data reduction: *SAINT*; program(s) used to solve structure: *SHELXS97* (Sheldrick, 2008 ▶); program(s) used to refine structure: *SHELXL97* (Sheldrick, 2008 ▶); molecular graphics: *PLATON* (Spek, 2009 ▶); software used to prepare material for publication: *SHELXTL* (Sheldrick, 2008 ▶).

## Supplementary Material

Crystal structure: contains datablocks global, I. DOI: 10.1107/S1600536809054828/lh2955sup1.cif

Structure factors: contains datablocks I. DOI: 10.1107/S1600536809054828/lh2955Isup2.hkl

Additional supplementary materials:  crystallographic information; 3D view; checkCIF report

## Figures and Tables

**Table 1:** Hydrogen-bond geometry (Å, °)

*D*—H⋯*A*	*D*—H	H⋯*A*	*D*⋯*A*	*D*—H⋯*A*
N1—H1*N*⋯O2^i^	0.87 (5)	1.85 (5)	2.72 (2)	170 (4)
N1—H1*N*⋯O2*A*^i^	0.87 (5)	1.92 (5)	2.73 (2)	154 (4)

Symmetry code: (i) 
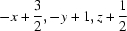
.

**Fig. 1.**
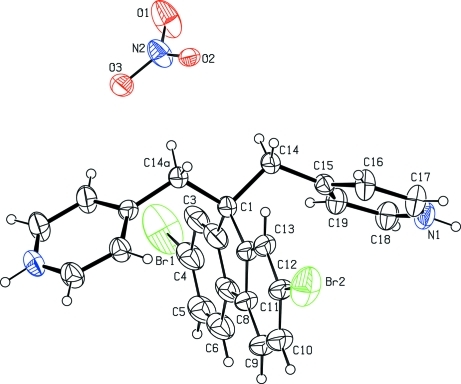
The molecular structure of the title compound showing 30% probability displacement ellipsoids. The disorder is not shown but thinner bonds show the areas where disorder occurs.

## supplementary crystallographic information

### Comment

Bipyridine compounds have been studied as spacer molecules both in organic and organic-inorganic hybrid complexes (Varughese & Pedireddi, 2005,2006) Their ability to form intermolecular hydrogen bonds is of particular interest (Pedireddi & Lekshmi, 2004; Friscic & MacGillivray, 2005). We present herein the crystal structure of the titlecompound. The asymmetric unit of the title compund is shown in Fig. 1. The cation moleclue lies on a twofold rotation axis about which the dibromofluorene moiety is disordered. Atom C1 lies on the twofold rotation axis. In addtion, the unique nitrate anion is disodered over two general sites with equall occupancies. The crystal structure is stabilized by intermoecular N-H···O hydrogen bonds.

### Experimental

To a warm solution of 2,7-dibromo-9,9-(4-pyridyl-methyl)fluorene [2.55 g, 5.0 mmol] in EtOH (50 ml), HNO_3_(10.0 mmol) was added dropwise with stirring. The mixture turned clear yellow. 1 h later, the yellow solution was filtered, and the filtrate was evaporated at room temperature in air. Three days later, crystals suitable for an X-ray structure determination were obtained.

### Refinement

H atoms were fixed geometrically and allowed to ride on their attached atoms, with C—H distances = 0.93-0.97 Å, and with *U*_iso_=1.2*U*_eq_(C). The H atom bonded to N1 was refined independently with an isotropic displacement parameter. The occupancies of the disorder components of the nitrate anion were intially refined but then fixed at 0.50:0.50.

### Crystal data

**Table:**

C_25_H_20_Br_2_N_2_^2+^·2NO_3_^−^	*F*(000) = 2528
*M**_r_* = 632.27	*D*_x_ = 1.568 Mg m^−^^3^
Orthorhombic, *F**d**d*2	Mo *K*α radiation, λ = 0.71073 Å
Hall symbol: F 2 -2d	Cell parameters from 2016 reflections
*a* = 14.874 (3) Å	θ = 3.3–27.9°
*b* = 33.592 (7) Å	µ = 3.07 mm^−^^1^
*c* = 10.720 (2) Å	*T* = 293 K
*V* = 5356.2 (18) Å^3^	Block, yellow
*Z* = 8	0.25 × 0.20 × 0.18 mm

### Data collection

**Table:**

Bruker SMART CCD diffractometer	3053 independent reflections
Radiation source: fine-focus sealed tube	1355 reflections with *I* > 2σ(*I*)
graphite	*R*_int_ = 0.065
φ scans and ω scans with κ offsets	θ_max_ = 27.5°, θ_min_ = 3.0°
Absorption correction: multi-scan (*SADABS*; Sheldrick, 1996)	*h* = −18→19
*T*_min_ = 0.514, *T*_max_ = 0.608	*k* = −43→42
12890 measured reflections	*l* = −13→13

### Refinement

**Table:**

Refinement on *F*^2^	Secondary atom site location: difference Fourier map
Least-squares matrix: full	Hydrogen site location: inferred from neighbouring sites
*R*[*F*^2^ > 2σ(*F*^2^)] = 0.041	H atoms treated by a mixture of independent and constrained refinement
*wR*(*F*^2^) = 0.114	*w* = 1/[σ^2^(*F*_o_^2^) + (0.0494*P*)^2^] where *P* = (*F*_o_^2^ + 2*F*_c_^2^)/3
*S* = 0.91	(Δ/σ)_max_ < 0.001
3053 reflections	Δρ_max_ = 0.20 e Å^−^^3^
238 parameters	Δρ_min_ = −0.18 e Å^−^^3^
77 restraints	Absolute structure: Flack (1983), 1443 Friedel pairs
Primary atom site location: structure-invariant direct methods	Flack parameter: 0.002 (14)

### Special details

**Table:**

Geometry. All esds (except the esd in the dihedral angle between two l.s. planes) are estimated using the full covariance matrix. The cell esds are taken into account individually in the estimation of esds in distances, angles and torsion angles; correlations between esds in cell parameters are only used when they are defined by crystal symmetry. An approximate (isotropic) treatment of cell esds is used for estimating esds involving l.s. planes.
Refinement. Refinement of F^2^ against ALL reflections. The weighted R-factor wR and goodness of fit S are based on F^2^, conventional R-factors R are based on F, with F set to zero for negative F^2^. The threshold expression of F^2^ > 2sigma(F^2^) is used only for calculating R-factors(gt) etc. and is not relevant to the choice of reflections for refinement. R-factors based on F^2^ are statistically about twice as large as those based on F, and R- factors based on ALL data will be even larger.

### Fractional atomic coordinates and isotropic or equivalent isotropic displacement parameters (Å^2^)

**Table:**

	*x*	*y*	*z*	*U*_iso_*/*U*_eq_	Occ. (<1)
Br1	0.5373 (4)	0.34967 (12)	1.0031 (6)	0.177 (2)	0.50
Br2	0.4644 (3)	0.66079 (12)	0.9441 (5)	0.1266 (12)	0.50
C1	0.5000	0.5000	0.8526 (6)	0.0493 (14)	
C2	0.5086 (13)	0.4687 (4)	0.9446 (10)	0.074 (3)	0.50
C3	0.5195 (12)	0.4279 (4)	0.9302 (8)	0.082 (3)	0.50
H3A	0.5215	0.4168	0.8508	0.099*	0.50
C4	0.5274 (9)	0.4036 (3)	1.0347 (11)	0.085 (3)	0.50
C5	0.5244 (7)	0.4202 (2)	1.1534 (9)	0.086 (3)	0.50
H5A	0.5297	0.4039	1.2233	0.103*	0.50
C6	0.5136 (7)	0.4610 (3)	1.1677 (9)	0.085 (3)	0.50
H6A	0.5116	0.4721	1.2472	0.101*	0.50
C7	0.5057 (10)	0.4853 (2)	1.0633 (12)	0.074 (3)	0.50
C8	0.4931 (10)	0.5280 (2)	1.0631 (10)	0.066 (2)	0.50
C9	0.4851 (7)	0.5582 (2)	1.1511 (8)	0.075 (2)	0.50
H9A	0.4857	0.5521	1.2357	0.090*	0.50
C10	0.4761 (7)	0.5975 (2)	1.1126 (8)	0.077 (2)	0.50
H10A	0.4708	0.6177	1.1715	0.093*	0.50
C11	0.4752 (8)	0.6066 (3)	0.9861 (8)	0.067 (2)	0.50
C12	0.4832 (11)	0.5765 (3)	0.8981 (7)	0.063 (2)	0.50
H12A	0.4826	0.5826	0.8135	0.075*	0.50
C13	0.4922 (12)	0.5371 (3)	0.9366 (9)	0.058 (2)	0.50
N1	0.8313 (2)	0.53409 (10)	0.9320 (5)	0.0682 (11)	
C14	0.58325 (19)	0.50368 (11)	0.7642 (5)	0.0510 (9)	
H14A	0.5919	0.4785	0.7217	0.061*	
H14B	0.5705	0.5237	0.7013	0.061*	
C15	0.6693 (2)	0.51471 (10)	0.8298 (4)	0.0467 (9)	
C16	0.7043 (2)	0.55303 (12)	0.8175 (6)	0.0694 (14)	
H16A	0.6722	0.5724	0.7744	0.083*	
C17	0.7869 (3)	0.56209 (14)	0.8696 (6)	0.0766 (15)	
H17A	0.8111	0.5875	0.8610	0.092*	
C18	0.8004 (2)	0.49768 (12)	0.9466 (6)	0.0690 (13)	
H18A	0.8341	0.4790	0.9900	0.083*	
C19	0.7187 (2)	0.48735 (11)	0.8980 (5)	0.0589 (11)	
H19A	0.6963	0.4618	0.9108	0.071*	
O1	0.5248 (7)	0.3942 (3)	0.4656 (13)	0.111 (4)	0.50
O2	0.4986 (12)	0.4542 (5)	0.5171 (11)	0.045 (3)	0.50
O3	0.4155 (14)	0.4101 (6)	0.5905 (15)	0.076 (4)	0.50
N2	0.4807 (2)	0.41997 (11)	0.52359 (13)	0.0733 (12)	
O1A	0.5534 (7)	0.4013 (4)	0.5514 (14)	0.151 (7)	0.50
O2A	0.4890 (13)	0.4546 (6)	0.4700 (11)	0.055 (3)	0.50
O3A	0.4017 (12)	0.4069 (6)	0.5454 (15)	0.060 (3)	0.50
H1N	0.883 (3)	0.5389 (11)	0.968 (5)	0.078 (13)*	

### Atomic displacement parameters (Å^2^)

**Table:**

	*U*^11^	*U*^22^	*U*^33^	*U*^12^	*U*^13^	*U*^23^
Br1	0.170 (2)	0.098 (2)	0.262 (6)	0.0198 (17)	0.038 (3)	0.097 (3)
Br2	0.1092 (12)	0.0779 (15)	0.193 (4)	0.0056 (9)	−0.0114 (15)	−0.0480 (15)
C1	0.037 (2)	0.063 (3)	0.048 (4)	−0.002 (2)	0.000	0.000
C2	0.050 (5)	0.108 (6)	0.064 (5)	0.002 (5)	0.008 (5)	0.028 (5)
C3	0.061 (5)	0.113 (6)	0.073 (6)	−0.001 (5)	0.012 (5)	0.034 (5)
C4	0.073 (5)	0.111 (5)	0.071 (6)	0.003 (5)	0.008 (5)	0.041 (5)
C5	0.079 (5)	0.109 (6)	0.070 (5)	0.002 (4)	0.004 (4)	0.034 (5)
C6	0.072 (4)	0.114 (6)	0.068 (5)	0.005 (5)	0.005 (4)	0.030 (5)
C7	0.052 (4)	0.111 (6)	0.057 (5)	0.001 (5)	0.004 (4)	0.032 (5)
C8	0.057 (4)	0.094 (5)	0.048 (5)	−0.007 (4)	0.002 (4)	−0.023 (4)
C9	0.073 (4)	0.097 (5)	0.055 (5)	0.000 (4)	0.005 (4)	−0.025 (4)
C10	0.074 (4)	0.098 (5)	0.060 (5)	0.001 (4)	0.003 (4)	−0.021 (4)
C11	0.060 (4)	0.085 (4)	0.056 (5)	0.001 (4)	0.003 (4)	−0.028 (4)
C12	0.052 (4)	0.078 (4)	0.058 (5)	−0.001 (4)	0.000 (4)	−0.019 (4)
C13	0.047 (4)	0.079 (4)	0.049 (5)	−0.002 (4)	0.002 (4)	−0.018 (4)
N1	0.0453 (18)	0.082 (3)	0.078 (3)	−0.0047 (17)	−0.0146 (19)	−0.004 (2)
C14	0.0398 (15)	0.064 (2)	0.049 (3)	−0.0031 (16)	0.0002 (16)	0.0011 (19)
C15	0.0409 (17)	0.0531 (19)	0.046 (3)	0.0005 (15)	0.0012 (17)	0.0044 (18)
C16	0.052 (2)	0.059 (2)	0.097 (4)	−0.0097 (17)	−0.015 (2)	0.016 (2)
C17	0.057 (2)	0.069 (3)	0.104 (5)	−0.0135 (19)	−0.020 (2)	0.004 (3)
C18	0.052 (2)	0.073 (3)	0.082 (4)	0.0064 (18)	−0.016 (2)	0.007 (3)
C19	0.049 (2)	0.056 (2)	0.072 (3)	0.0011 (16)	−0.0079 (19)	0.003 (2)
O1	0.090 (8)	0.061 (4)	0.182 (13)	0.012 (5)	0.057 (7)	0.000 (7)
O2	0.055 (6)	0.045 (4)	0.036 (8)	−0.003 (3)	0.001 (6)	−0.004 (6)
O3	0.085 (9)	0.075 (5)	0.068 (12)	−0.030 (5)	0.023 (7)	−0.023 (8)
N2	0.061 (2)	0.063 (3)	0.096 (4)	0.0058 (19)	0.029 (2)	0.002 (2)
O1A	0.064 (5)	0.102 (8)	0.29 (2)	0.016 (5)	0.025 (8)	0.086 (11)
O2A	0.049 (4)	0.072 (5)	0.043 (9)	−0.002 (3)	−0.004 (6)	0.012 (7)
O3A	0.051 (5)	0.078 (6)	0.051 (9)	−0.013 (4)	0.005 (5)	−0.018 (6)

### Geometric parameters (Å, °)

**Table:**

Br1—C4	1.849 (9)	C11—C12	1.3900
Br2—C11	1.881 (9)	C12—C13	1.3900
C1—C2^i^	1.446 (9)	C12—H12A	0.9300
C1—C2	1.446 (9)	N1—C18	1.316 (5)
C1—C13	1.543 (8)	N1—C17	1.330 (5)
C1—C13^i^	1.543 (9)	N1—H1N	0.87 (5)
C1—C14^i^	1.564 (5)	C14—C15	1.506 (5)
C1—C14	1.564 (5)	C14—H14A	0.9700
C2—C3	1.3900	C14—H14B	0.9700
C2—C7	1.3900	C15—C19	1.386 (5)
C3—C4	1.3900	C15—C16	1.395 (5)
C3—H3A	0.9300	C16—C17	1.384 (6)
C4—C5	1.3900	C16—H16A	0.9300
C5—C6	1.3900	C17—H17A	0.9300
C5—H5A	0.9300	C18—C19	1.366 (5)
C6—C7	1.3900	C18—H18A	0.9300
C6—H6A	0.9300	C19—H19A	0.9300
C7—C8	1.447 (9)	O1—N2	1.252 (11)
C8—C9	1.3900	O2—N2	1.182 (19)
C8—C13	1.3900	O3—N2	1.25 (2)
C9—C10	1.3900	N2—O3A	1.28 (2)
C9—H9A	0.9300	N2—O1A	1.284 (11)
C10—C11	1.3900	N2—O2A	1.30 (2)
C10—H10A	0.9300		
			
C2^i^—C1—C2	94.1 (12)	C11—C12—C13	120.0
C2—C1—C13	101.3 (5)	C11—C12—H12A	120.0
C2^i^—C1—C13^i^	101.3 (6)	C13—C12—H12A	120.0
C13—C1—C13^i^	108.6 (13)	C12—C13—C8	120.0
C2^i^—C1—C14^i^	113.6 (7)	C12—C13—C1	127.0 (7)
C2—C1—C14^i^	115.2 (7)	C8—C13—C1	112.9 (7)
C13—C1—C14^i^	111.0 (7)	C18—N1—C17	122.9 (4)
C13^i^—C1—C14^i^	110.4 (11)	C18—N1—H1N	115 (3)
C2^i^—C1—C14	115.2 (7)	C17—N1—H1N	122 (3)
C2—C1—C14	113.6 (7)	C15—C14—C1	114.1 (4)
C13—C1—C14	110.4 (6)	C15—C14—H14A	108.7
C13^i^—C1—C14	111.0 (11)	C1—C14—H14A	108.7
C14^i^—C1—C14	105.4 (5)	C15—C14—H14B	108.7
C3—C2—C7	120.0	C1—C14—H14B	108.7
C3—C2—C1	130.7 (9)	H14A—C14—H14B	107.6
C7—C2—C1	109.3 (9)	C19—C15—C16	117.6 (3)
C2—C3—C4	120.0	C19—C15—C14	122.3 (3)
C2—C3—H3A	120.0	C16—C15—C14	120.0 (3)
C4—C3—H3A	120.0	C17—C16—C15	119.8 (4)
C3—C4—C5	120.0	C17—C16—H16A	120.1
C3—C4—Br1	115.7 (7)	C15—C16—H16A	120.1
C5—C4—Br1	124.2 (7)	N1—C17—C16	119.2 (4)
C6—C5—C4	120.0	N1—C17—H17A	120.4
C6—C5—H5A	120.0	C16—C17—H17A	120.4
C4—C5—H5A	120.0	N1—C18—C19	120.1 (4)
C7—C6—C5	120.0	N1—C18—H18A	119.9
C7—C6—H6A	120.0	C19—C18—H18A	119.9
C5—C6—H6A	120.0	C18—C19—C15	120.3 (3)
C6—C7—C2	120.0	C18—C19—H19A	119.8
C6—C7—C8	126.5 (11)	C15—C19—H19A	119.8
C2—C7—C8	113.5 (11)	O2—N2—O3	117.7 (13)
C9—C8—C13	120.0	O2—N2—O1	121.8 (11)
C9—C8—C7	137.2 (10)	O3—N2—O1	120.5 (11)
C13—C8—C7	102.8 (10)	O2—N2—O3A	123.6 (14)
C10—C9—C8	120.0	O1—N2—O3A	109.5 (9)
C10—C9—H9A	120.0	O2—N2—O1A	107.4 (9)
C8—C9—H9A	120.0	O3—N2—O1A	113.0 (11)
C9—C10—C11	120.0	O3A—N2—O1A	124.4 (11)
C9—C10—H10A	120.0	O3—N2—O2A	124.2 (15)
C11—C10—H10A	120.0	O1—N2—O2A	110.5 (10)
C12—C11—C10	120.0	O3A—N2—O2A	118.4 (13)
C12—C11—Br2	123.4 (5)	O1A—N2—O2A	117.2 (11)
C10—C11—Br2	116.6 (5)		

Symmetry codes: (i) −*x*+1, −*y*+1, *z*.

### Hydrogen-bond geometry (Å, °)

**Table:**

*D*—H···*A*	*D*—H	H···*A*	*D*···*A*	*D*—H···*A*
N1—H1N···O2^ii^	0.87 (5)	1.85 (5)	2.72 (2)	170 (4)
N1—H1N···O2A^ii^	0.87 (5)	1.92 (5)	2.73 (2)	154 (4)

Symmetry codes: (ii) −*x*+3/2, −*y*+1, *z*+1/2.
